# Reversible Photoalignment of Liquid Crystals: a Path toward the Creation of Rewritable Lenses

**DOI:** 10.1038/s41598-020-62778-2

**Published:** 2020-04-01

**Authors:** Juan Antonio Quiroga, Ignacio Canga, José Alonso, Daniel Crespo

**Affiliations:** 1 Indizen Optical Technologies S.L., Parque Científico de Madrid, Faraday 7, Madrid, 28049 Spain; 2 Complutense University of Madrid, Optics Department, Physics School, Madrid, 28040 Spain

**Keywords:** Materials for optics, Liquid crystals

## Abstract

In this work, we describe a new reversible photoalignment effect for the director in nematic liquid crystals that provides an approach for the creation of lenses whose optical power can be recorded and erased. The possibility of creating a rewritable lens has very important practical implications, for example, in the ophthalmic lens industry. A rewritable ophthalmic lens could be a convenient solution for patients whose compensation needs change over time due to age-related physiological changes, such as the onset and progression of presbyopia. Using rewritable lenses, small lens power corrections could be implemented through a rewriting procedure on the mounted lens without resurfacing or manufacturing and mounting a new lens. More generally, this new effect multiple potential applications in the creation of rewritable optical systems, such as reconfigurable optical networks, index-tunable antireflective coatings and optically rewritable phase gratings.

## Introduction

Adaptive optical elements whose refractive state can be changed are of great interest in many industrial and scientific applications. A typical application example is a cellphone autofocus objective, for which the focal distance can be continuously changed, allowing for the imaging of objects at variable distances. In the case of a single lens, the power depends on the surface geometry and the refractive index. Spatial variation in the refractive index can be used in the same way as surface curvature to alter the optical power; such lenses are known as gradient index (GRIN) lenses. In the paraxial approximation, a thin slab of material with thickness *d* and a refractive index distribution *n*(*x*, *y*) has an optical power specified by the dioptric power matrix^1^, ${\mathbb{P}}$, given by

1 $${\mathbb{P}}(x,y)=-\,d{\mathbb{H}}[n(x,y)]$$

where ${\mathbb{H}}[n]$ is the Hessian matrix of *n*(*x*, *y*). For example, a circular flat lens with a diameter of 20 mm, a thickness *d* = 100 *μm* and a parabolic index distribution $n=0.002({x}^{2}+{y}^{2})+1.5$ behaves as a monofocal lens with a focal length of −2.5 *m* and a power of −0.4 D. More complex power distributions, such as those in progressive power lenses used for compensation of presbyopia, can also be achieved with GRIN materials^2–4^.

Liquid crystals are the best-known material for the creation of adaptive GRIN lenses^3^. The liquid crystal anisotropy permits the easy creation of refractive index profiles in a plano-parallel cell (a GRIN lens) through local director orientation. Current solutions use a local electric or magnetic field to modulate the local director orientation^3^. However, as soon as the external field disappears, the local director reverts to its equilibrium state, and the GRIN lens is erased. Therefore, current adaptive liquid crystal GRIN lenses require continuous power consumption to maintain the refractive state.

In this work, we describe a new reversible photoalignment effect of the director of a nematic liquid crystal mixture in homogeneous planar cells that provides an approach for the creation of rewritable GRIN lenses that can be recorded and erased. With some commercial liquid crystal mixtures, the creation of rewritable GRIN lenses is possible, where the power is written through exposure to unpolarized UV light and thermally erased, allowing for a further rewriting process. Once recorded, the GRIN lenses are stable for several days without the application of any external energy source.

The possibility of creating a stable rewritable GRIN lens has important practical implications, for example, in the ophthalmic lens industry. A rewritable ophthalmic lens could be a perfect solution for patients with evolving compensation needs due to age-related physiological changes, such as the onset and progression of presbyopia. Power correction could be implemented on the frame without the need for new lenses. Similarly, stock lenses could be manufactured such that the final prescription could be written once the lens is glazed and ready for mounting.

More generally, this new effect has multiple potential applications in the creation of rewritable optical systems, such as reconfigurable optical networks, index-tunable antireflective coatings, and optically rewritable phase gratings

## Exposure-Induced Polar Photoalignment

**Figure 1:**
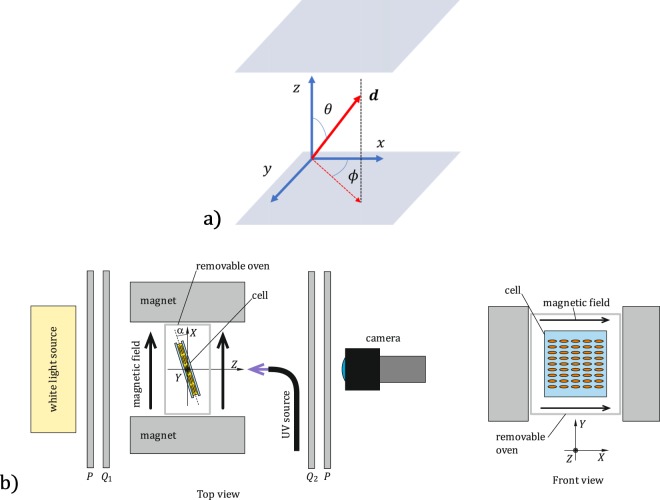
(**a**) Coordinate system for the polar (*θ*) and azimuthal (*ϕ*) angles that determine the director (***d***) orientation with respect the substrate normal, which, in our case, is aligned with the *z* axis. The *xy* plane is defined by the substrate plane of the cell. (**b**) Experimental setup used for the measurement of the polar photoalignment; the plano-parallel liquid crystal cell is placed between the $\lambda /4$ retarders of the polariscope, ${Q}_{1,2}$. The polariscope also uses a polarizer *p* before *Q*_1_ and an analyzer *A* after *Q*_2_. The sample is placed so that a small angle, *α*, is formed with the magnetic field to avoid the appearance of disclination walls. The extended white light source illuminates the sample, and the camera images the plane of the cell. The cell is UV irradiated normally by a light guide pointing toward the sample.

The simplest structure for studying polar photoalignment is the plano-parallel cell, for which the liquid crystal material is confined between two flat transparent plates; see Fig. 1a. Without external agents, such as an electric or magnetic field, the orientation of the molecules inside the cell will be in the equilibrium state, and in the nematic state, this orientation determines the effective index of refraction and therefore the refractive state of the cell.

In uniaxial liquid crystals, the director is defined as the spatiotemporal average of the direction of the optical axis of each molecule. The director concept can be used for liquid crystals and for mixtures. The orientation of the liquid crystal director inside the cell is surface mediated and depends on the azimuthal and polar anchoring energies of the liquid crystal on the surface. In the absence of an external agent, the interaction between the surface and the liquid crystal imposes a preferred direction on the director in the equilibrium state, known as the easy direction. The director is specified by its polar and azimuthal angles, which, in a plano-parallel cell, are determined with respect to the substrate’s normal. Following Fig. 1a, this normal will be parallel to the *z* axis. As we show in Fig. 1a, the polar angle *θ*, or pretilt, is given by the angle between the director ***d*** and the *z* axis. The angle between the director’s projection on the *xy* plane and the *x* axis is called the azimuthal angle, *ϕ*. The equilibrium pretilt is usually fixed using alignment layers on the cell substrates. For example, alignment layers of poly(methyl methacrylate) (PMMA), polyvinyl alcohol (PVA) and some types of polyimide (PI) allow for the creation of homogeneous planar cells with pretilt *θ* = 90°, while other PI alignment layers create homeotropic cells with pretilt *θ* = 0.

Many techniques can be used to control the easy direction in nematic liquid crystal cells. Among them, photoalignment methods^5,6^ are clearly advantageous because of their noncontact nature and, in some cases, reversibility. Additionally, they potentially allow for the modulation of the easy direction once the cell is assembled. Photoalignment techniques for control of the easy direction include the use of dye-doped alignment layers^7,8^ photodimerization^9^, photocrosslinking^10^ and photodegradation^11^. However, only the photoisomerization process, with the use of azo-dyes^7,12,13^ allows for a rewritable scheme. For example, in the case of dye-doped command surfaces, the azimuthal angle can be tuned or the polar angle switched between homogeneous and homeotropic conditions. Additionally, photoisomerization can induce a reversible nematic to isotropic phase transition in guest/host mixtures with a photoisomerizable dye.

However, only the reversible photoalignment of the director’s polar angle allows for the creation of rewritable GRIN lenses. In a plano-parallel nematic liquid crystal cell with a uniaxial material, the effective refractive index, *n*_*eff*_, perceived by a linearly polarized beam propagating along the *Z* axis with the same azimuthal angle as the local director depends only on the director’s polar angle and is given by

2 $$\frac{1}{{n}_{eff}^{2}(\theta )}=\frac{{\sin }^{2}\theta }{{n}_{e}^{2}}+\frac{{\cos }^{2}\theta }{{n}_{o}^{2}}$$

where *θ* is the polar angle and *n*_*o*,*e*_ are the ordinary and extraordinary refractive indexes of the liquid crystal. Therefore, for a polar angle distribution, *θ*(*x*,*y*), the resulting refractive index, *n*_*eff*_(*x*,*y*), given by Eq. (2) will generate a GRIN lens with paraxial power given by Eq. (1)^1,2^

In this work, we report a new photoalignment property of certain liquid crystal mixtures that allows for the reversible polar photoalignment of the director in a homogeneous liquid crystal cell. For liquid crystal mixtures presenting this property, the polar angle can be continuously tuned from *θ* = π/2 in the starting homogenous planar configuration to *θ* = 0 to realize the homeotropic condition. For a given liquid crystal, alignment layer, and cell thickness, the exposure to unpolarized UV light controls the resulting polar angle. For this reason, we have denominated this property exposure-induced polar photoalignment (EPPA). For the materials we discuss in this work, the polar photoalignment can be reversed to the original homogenous planar configuration by applying heat for a certain amount of time, allowing for further polar photoalignment. Due to the dependence of the effective refractive index on the polar angle, the EPPA effect provides an approach for the creation of rewritable GRIN optical elements for which the optical power can be written using an unpolarized UV beam and erased by heating the element, reverting the sample to its initial state.

To characterize the EPPA effect, we used the setup shown in Fig. 1b, where we depict a top view. A homogeneous cell is placed inside a circular polariscope^14^ with a configuration consisting of a polarizer, two quarter-wave plates and a final polarizer, usually denominated the analyzer. The sample is placed between the quarter-wave plates as shown in Fig. 1b, and we use a white light fluorescent source that behaves as a tricolor source with ${\lambda }_{B}=438\,{\rm{nm}},\,{\lambda }_{G}=548\,{\rm{nm}}$ and ${\lambda }_{R}=613\,{\rm{nm}}$ for the blue, green and red wavelengths, respectively. The cell is inserted in a magnetic field created by two Nd magnets oriented along the *X* axis. The sample is imaged by an RGB color camera.

The first example of a liquid crystal material with the EPPA property that we found was a commercially available mixture from Merck, named MLC-2132. This material is a complex mixture with a clearing point at *T*_*c*_ = 114 °C and refractive indexes *n*_*e*_ = 1.746 and *n*_*o*_ = 1.509 at *λ*_*R*_ (Δ*n* = 0.237). To test the EPPA property in MLC-2132, we used homogenous planar cells made with quartz plano-parallel plates and PMMA as an alignment layer.

Before we began the writing process, we orientate the cell normal along the *Z* axis (*α* = 0 in Fig. 1b), and the director was aligned azimuthally along the *X* axis by heating the sample at 85 °C in a magnetic field^15^. Our Nd magnets had a surface field of 524 mT. The gap between magnets was 39 mm, generating a magnetic field of 320 mT at the cell center. After this process, the liquid crystal molecules are aligned and anchored simultaneously on the PMMA alignment layer coating the quartz substrates. Therefore, before the exposure to UV radiation the director azimuthal and polar angles where *ϕ* = 0 and *θ* = 90° respectively.

Once the cell was aligned azimuthally along the *X* axis, we illuminated the sample with a UV beam generated by a plasma light source (HPLS343 from Thorlabs) filtered with a bandpass filter centered at 325 nm (FGUV11M from Thorlabs). The assembly behaved as a bandpass UV source centered at 370 nm with a spectral width of 30 nm and large tails below 360 nm. The source was connected to a 3 mm water light guide, whose output was concentrated on the sample with a 16 mm focal length aspherical lens.

**Figure 2:**
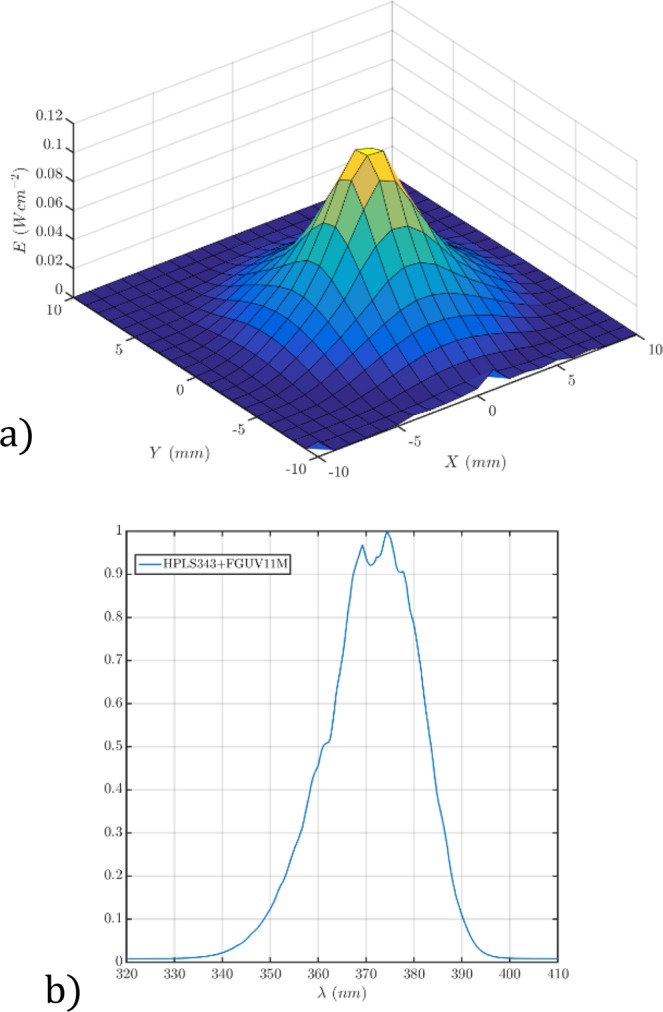
(**a**) 2D profile of the UV irradiance pattern at the cell plane. (**b**) Spectrum of the plasma light source filtered with the passband filter that illuminates the sample.

Figure 2a shows the UV irradiance profile used in our experiments. The normalized power spectrum of the plasma light source filtered by the UV bandpass filter is shown in Fig. 2b.

In our experiment, the polariscope elements of Fig. 1b were arranged in the circular dark field (CDF) configuration^14^, for which the polarizer and first quarter-wave plate were set at 45°, as were the second quarter-wave plate and the analyzer, effectively forming two crossed circular polarizers with the sample placed between them. The sample was irradiated at intervals of Δ*t* seconds, and we captured a CDF image after each exposure. The use of a color camera with a white tricolor illuminating lamp is very convenient for observing optical retardation as color changes in the RGB image. To measure the retardation, we used the red band that corresponds to a monochromatic image with wavelength *λ*_*R*_. In the CDF configuration, for each (*x*,*y*) point on the sample, the temporal intensity transmitted by the polariscope at time *t*_*k*_ = *k*Δ*t* is given by^14^.

3 $$I(k)=\frac{{I}_{0}}{2}[1-\,\cos \,\delta (k)],$$

where *I*_0_ is the lamp intensity. For a given point, each temporal sample, *I*(*k*), corresponds to an exposure *H*(*k*) = *k*Δ*tE*, where *E* is the incident irradiance at the point of interest. From Eq. (3), the recorded intensity at each point is a temporal interferogram with a monotonic decreasing phase given by the optical retardation

4 $$\delta (k)=\frac{2\pi }{{\lambda }_{R}}{\int }_{0}^{d}[{n}_{eff}(k,z)-{n}_{o}]dz,$$

where *d* is the cell thickness, *λ*_*R*_ is the wavelength, *n*_*eff*_(*k*,*z*) is the spatial distribution along the *z* axis of the effective extraordinary index, and *n*_0_ is the ordinary index. If the cell is in a homogenous planar condition with the director aligned along the *X* axis (polar angle *θ* = π/2, then *n*_*eff*_ (*z*) = *n*_*e*_, and the retardation reaches its maximum value ${\delta }_{M}=\frac{2\pi }{\lambda }d\Delta n$. In contrast, if the polar angle becomes *θ* = 0 for the whole cell thickness, then *n*_*eff*_(*z*) = *n*_0_, the retardation becomes zero and the alignment condition will be homeotropic.

In all our experiments, the director was initially horizontal, that is, $\theta (k=0)=\pi /2$, Therefore, the rotation direction was indeterminate, clockwise or counterclockwise, as the polar angle starts changing with the incident UV exposure. In other words, for two close locations, the generated pretilts could have opposite directions, producing disclination walls^16,17^ This phenomenon may also occur between the two substrates of the cell^18^. To break this symmetry, we rotated the sample around the *y* axis by α = 10° with respect to the magnetic field. In this way, we set a preferred rotation direction for the polar angle for both cell substrates. In addition, the magnetic field maintained the director’s azimuthal orientation along the *X* axis during the recording and erasing process.

To demonstrate the EPPA effect in MLC-2132, we recorded/erased a retardation pattern three times using the UV irradiance pattern shown in Fig. 2a. For all irradiation cycles, we used intervals of $\Delta t=15\,{\rm{s}}$ and recorded a temporal set of CDF images given by Eq. (3). For each cycle, the temporal variation in the optical retardation $\delta (k)$ at a point was obtained from the temporal signal *I*(*k*) using a Fourier transform temporal demodulation technique^19^.

**Figure 3:**
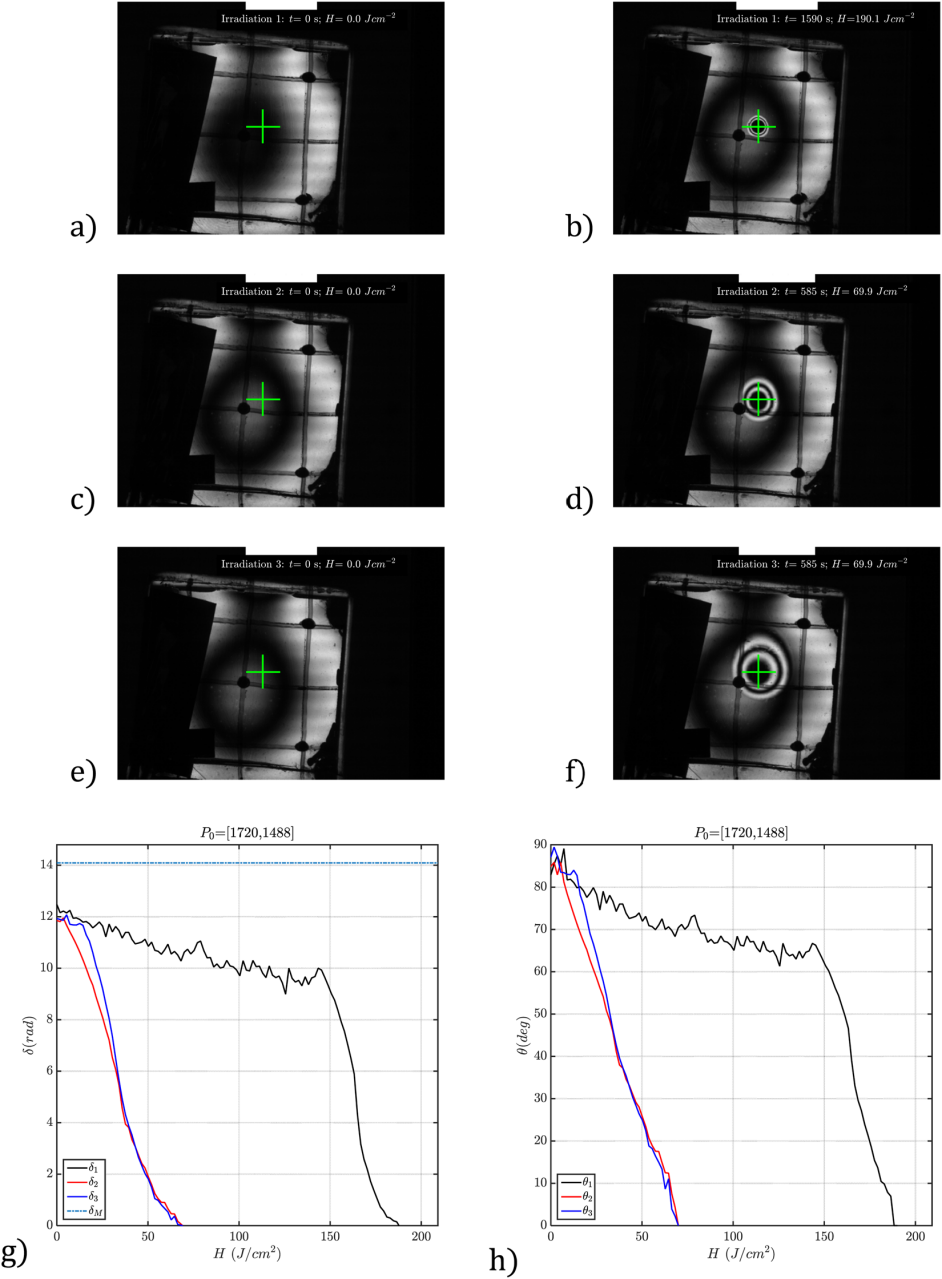
Recording-erasing CDF images for a 5.8 *μm* cell. (**a**) Initial state of the cell, and (**b**) first recording. Note the circular fringe pattern at the beam incidence point indicated by the green cross. The black disk indicates a homeotropic region. (**c**) CDF image of the cell after the first erasing operation. (**d**) Image of the cell after the second writing cycle; as in the first cycle, we reached the homeotropic state at the center of the fringe pattern, and the retardation slowly increases toward the edges. (**e**,**f**) Cell state after the second erasure and the third recording. For better visualization of the retardation evolution for the three recording cycles, You can find here a time-lapse movie clip with the temporal evolution of the CDF fringe patterns shown in figures (**a**) to (**f**). (**g**) Retardation measurement as a function of the exposure at the point marked by the green cross in Fig. 3. *δ*_1_ (black), *δ*_2_ (red) and *δ*_3_ (blue) plot the three measured retardation curves as a function of the exposure *H*. As a reference, we also show the value for the maximum retardation available, ${\delta }_{M}=\frac{2\pi }{\lambda }d\Delta n=14.1$ rad. h) *θ*_1_ (black), *θ*_2_ (red) and *θ*_3_ (blue) plot the polar angle for the three retardation curves of (**g**) assuming that the polar angle is uniform across the cell section.

In Fig. 3a,b, we show the initial and final states for the first recording cycle. In both figures, we can see the square cell with ink markings for spatial registration of the incident beam. The dark polygonal region to the left side of the cell is a fixture used to attach the thermocouple used to control the temperature during the erasing process. The green cross indicates the center of the incident irradiance pattern. The thickness measured at the cell center before filling was $d=5.8\,\mu m$. The total irradiance time in the first cycle was ${t}_{1}=1590\,s$ after 106 exposures of 15 s each. The irradiance of the writing beam at the point of interest was $E=115\,{\rm{mW}}/{{\rm{cm}}}^{2}$, and the accumulated exposure was ${H}_{1}=183\,{\rm{J}}/{{\rm{cm}}}^{2}$. Figure 3b shows that the central part of the CDF pattern is dark at the center, indicating a homeotropic condition for which the polar angle is *θ* = 0 and *n*_*eff*_ = *n*_0_. The homeotropic condition was confirmed with a conoscopic measurement at the central dark fringe marked with the green cross and through the observation of the sample inside the polariscope at different angles.

In Fig. 3g, we depict the measured retardations as a function of the exposure. The black line (labeled *δ*_1_) corresponds to the temporal retardation for the first recording cycle. The retardation continuously changes from its maximum value to zero. Initially, the cell was in a homogeneous state with the director azimuthally aligned along the *X* axis, and over the recording, this alignment was maintained. Therefore, the retardation change we observe is generated by polar photoalignment of the local director between 90° and 0°, which produces a continuous change in the effective refraction index. As discussed above, this effect is what we call exposure-induced polar photoalignment (EPPA). If we assume that the pretilt is constant along the Z axis for the entire cell thickness, from Eq. (4), each fringe in the CDF image of Fig. 3b represents a contour line of *n*_*eff*_ with a nominal variation of $\Delta {n}_{eff}={\lambda }_{R}/d\approx 0.1$ for the cell thickness $d=5.8\,\mu m$. As a reference, in Fig. 3g, we also plot the nominal value for ${\delta }_{M}=\frac{2\pi }{{\lambda }_{R}}d\Delta n=14.1\,{\rm{rad}}$ for this cell. We say nominal because the thickness was measured in air, and after the filling, the cell thickness at the center may change. As we will show, the refractive index distribution remains stable for days, providing the possibility to record refractive index distributions in liquid crystal cells. In the supplementary material, note 3, we present a −1 *D* GRIN lens recorded in a 51 *μm* MLC-2132 cell. The photoinduced retardation was very stable for two days and after 7 days the maximum retardation has dropped by 45% of its initial value.

In general, the polar angle will have a non-uniform value trough the cell thickness, but it can be very relevant. For informative purposes, in Fig. 3h, we have plot the polar angle for the three retardation curves of Fig. 3g using Eqs. (2) and (4) and assuming that the polar angle is uniform across the cell section.

After this first irradiation cycle, we proceeded with the erasing phase. For this, we heated the cell *in situ* by means of an oven for 20 h at *T*_*e*_ = 85 °C while keeping the sample inside tilted by 10° relative to the magnetic field. The erasing oven was manufactured using ITO-coated glass slides to allow for *in situ* observation while the cell was erased. The magnetic field was used in this case to help maintain the azimuthal orientation and ensure proper polar angle reversal. The erasing temperature *T*_*e*_ = 85 °C is the same temperature used for azimuthal alignment with a magnetic field. We chose this erasing temperature because at this value starts the surface melting process of the PMMA layer^15^ that eventually ends with its total degradation. Additionally, at this temperature, the MLC-2132 mixture is in the nematic phase (*T*_*c*_ = 114 °C). After this erasing process, the director’s polar angle became 90°, and the cell reverted to the initial homogenous planar condition. A CDF image of the cell after erasure is shown in Fig. 3c. The recorded pattern shown in Fig. 3b was erased, recovering the retardation map of Fig. 3a.

After the first erasure, we proceeded with a new recording cycle using the same parameters as before. For this second process, the number of 15 s exposures needed to reach the homeotropic condition was 39, with accumulated time and exposure of ${t}_{2}=585\,{\rm{s}}$ and ${H}_{2}=67.2\,{{\rm{Jcm}}}^{-2}$, respectively. In Fig. 3d, we show a CDF image of the cell at the end of the second recording cycle. In Fig. 3g, the red continuous line (labeled ${\delta }_{2}$) shows the retardation for the second recording cycle. The exposure necessary to reach the homeotropic state is approximately one-third that in the first cycle. This second recording cycle ${\delta }_{2}(H)\,$is clearly different from ${\delta }_{1}(H)$.

From Fig. 3g, the first recording process consists of two regimes. In the first regime (which spans from approximately 0 to 140 ${{\rm{Jcm}}}^{-2}$), the retardation ${\delta }_{1}$ slowly changes from 12 rad to 10 rad. If we assume a uniform polar angle along the cell thickness, this retardation change represents a variation of 30° in the pretilt. From 140 ${{\rm{Jcm}}}^{-2}$ to ${H}_{1}=182.85\,{{\rm{Jcm}}}^{-2}$, a second recording regime appears in which the retardation - and the polar angle - changes faster with the exposure. The transition between the two regimes is marked by a well-distinguishable elbow or corner in the ${\delta }_{1}(H)$ curve.

In the case of the second cycle, after the erasure, the first recording regime disappears, no elbow is observed, and only the second and faster regime appears. This behavior is confirmed by applying a third erasing-recording cycle, again with the same parameters as above. In Fig. 3g, we show in blue (labeled ${\delta }_{3}$) the third retardation recording, which fairly matches ${\delta }_{2}(H)$. Figure 3e,f show the starting and final CDF images of the third recording cycle. As in the case of the second cycle, the time necessary to reach the homeotropic condition was ${t}_{3}=585\,s$, and the accumulated exposure was ${H}_{3}=67.2\,Jc{m}^{-2}$. Therefore, with 20 h of erasure at 80 °C, a change occurs in the EPPA dynamics that is expressed as the disappearance of the first recording regime and the elbow in ${\delta }_{1}(H)$ for the $5.8\,\mu m$ thick MLC-2132 cell. For better visualization of the retardation evolution for the three recording cycles, You can find here a time-lapse movie clip with the temporal evolution of the circular dark field fringe pattern.

Importantly, during the recording-erasing cycles shown in Fig. 3, the mixture is in the nematic phase, and the azimuthal angle is kept constant; therefore, the retardation changes we observe are due only to polar angle variation.

## Discussion

All our recording-erasing retardation experiments indicate that EPPA is surface mediated by a UV-induced change in the liquid crystal mixture. For thin cells (up to $15\,{\rm{to}}\,20\,{\rm{\mu }}{\rm{m}}$), total reorientation between homogeneous and homeotropic conditions is possible. For thicker cells, we obtain similar results, but total reorientation of the liquid crystal director along the cell thickness can be hindered (see supplementary material note 1). This result is a strong indicator that the EPPA effect is surface mediated and works more efficiently in thin cells, where the cell volume has less importance with respect to the surfaces. Additionally, the total exposure at the cell plane controls the EPPA effect. The recording beam directionality spatial structure is not important for the polar photoalignment (see supplementary material note 2).

Inspection of Fig. 3b,d, and 3f shows that the size of the recorded fringe pattern associated with the extraordinary refractive index profile grows in diameter with the recording cycle. The origin of this behavior is mainly due to the elastic, non-local, nature of the nematic liquid crystal matrix. The collective nature of the liquid crystal matrix combined with small changes in the initial retardation state between recording cycles can produce significant variations far from the irradiation point in the recorded polar angle spatial distribution. For example, in Fig. 3 the variation in the initial retardation state after each erasing step can be perceived in Fig. 3a,c,e as a slight change in the central circular fringe.

In practical terms, the non-local behavior of the nematic matrix makes very difficult the reproducible recording of a spatial GRIN profile with a constant-power UV beam. However, controlling the power of the UV beam spatially and monitoring the generated retardation, it is possible to reach the desired retardation state in a reproducible way.

Although we obtained the results of this work using the MLC-2132 mixture, we observed that the MLC-2171 and MLC-2172 mixtures from Merck also present the EPPA effect at the same wavelengths. All these MLC mixtures are complex products with more than 10 components that may interact in different ways with UV radiation. In our tests for the EPPA effect, high birefringence and high clearing point are not necessary conditions and the nature of the materials is very relevant. For example, we tested with negative results the mixtures QYPDLC-142 and QYTN-82 from Qingdao QY Liquid Crystal Co., Ltd that have with similar physical characteristics than the MLC mixtures mentioned above.

Currently, our main working hypothesis is that UV radiation interacts with at least one of the components in the MLC mixture, producing a stable photoisomer that modifies the polarity of the mixture, changing the equilibrium polar angle at the alignment layer interface. Then, depending on the photoisomer concentration, the pretilt continuously changes from 90 to 0 deg without breaking the nematic phase. The local order of the nematic phase slows the diffusion of the photoisomer, allowing for stable polar angle distributions at the two interfaces of the cell that in turn induce a volume orientation of the polar angle. With respect to temporal stability, our recorded index distributions can last for several days at room temperature (see supplementary material note 3). Additionally, we observed a spectral dependence of the EPPA effect. Although the efficiency of the process was better at 320 nm, in our case, the best compromise between the radiant flux and efficiency was the use of a 365 nm wavelength for the UV recording beams (see supplementary material note 4).

Following our hypothesis, the explanation for the erasing mechanism is that the UV-generated photoisomer reverts with application of thermal energy. This phenomenon is supported by the rewritable character of EPPA shown in Fig. 3g.

An alternative mechanism for the recording/photoalignment could be photodegradation of the alignment layer with the UV radiation. However, this process should not be reversible and would not allow for the thermal erasing process. Additionally, EPPA does not depend on the alignment layer as long as the starting alignment configuration is homogeneous (see supplementary material note 2). In our experiments, we also observed a yellowing of the samples that suggests a photodegradation process; however, the thermal reversibility is a good indicator that the yellowing is not connected with the EPPA effect and may be produced by another component of the mixture.

In summary, in this work, we report a new polar photoalignment mechanism in nematic liquid crystal mixtures, the EPPA effect. This new mechanism allows for the creation of stable refractive index distributions using unpolarized UV light. The recorded refractive index distributions can be erased by heating the sample. EPPA provides a potential approach for creating GRIN lenses for which the refractive state can be adapted over time to evolving needs.

Moreover, the EPPA effect can also be used to generate optical elements such as retardation plates, aspherical optics, polarization diffraction gratings or special geometry microlens arrays that otherwise can be very difficult to generate using conventional manufacturing methods.

The single cell configuration allows for the creation of devices that work only with polarized light. More general polarization-independent configurations can be devised using well-known techniques^20^.

## Methods

### Retardation measurement

The motorized polariscope we used for capturing the CDF images was a diffuse light circular polariscope with a stepper motor and a 340 mm diameter field of view from Tiedemann instruments GmbH, model AE131. As an extended white light source for the polariscope, we used an array of 4 cool white fluorescent lamps, model Dulux 840 14 W, from Osram with a color temperature of 4000 K.

For the azimuthal alignment, the magnets we used were axially magnetized Nd blocks of 44 × 45 × 30 mm from AimanGz, model NB045.

The camera in the polariscope setup was a DFK33UJ003 CMOS camera from The Imaging Source GmbH.

For local measurements of retardation in liquid crystal cells, we used a BX53M polarizing microscope from Olympus equipped with a model U-CTB thick Berek compensator that can measure retardation up to 20 fringes at 550 nm. For the MLC mixtures, compensation of the different material dispersions between the LC mixtures and the compensator crystal was necessary^21^.

With the temporal demodulation, we have 4% uncertainty in the retardation measurement. In this case, it is very important to assure that the temporal interferogram (see Eq. 3) has at least 4–8 samples per cycle. Also, we use a 3 × 3 spatial averaging on each frame to reduce the temporal signal noise.

For the Berek compensator, taking into account the liquid crystal dispersion, we achieve a 3% uncertainty.

### Liquid crystal mixtures

In our experiments, we used the MLC-2132, MLC-2172 and MLC-2171 mixtures from Merck. Also, we tested for EPPA with negative results the QYTN-802 and QYPDLC-142 from Qingdao QY Liquid Crystal Co. These materials are high birefringence and high clearing point complex mixtures with many components. In Table 1, we summarize their birefringence and clearing points, obtained from the Merck and Qingdao datasheet.

**Table 1:** Birefringence and clearing points for the liquid crystal mixtures that we have tested for the EPPA property.

Mixture	Δ*n*(@589.3 *nm*)	*T*_*c*_(C)
MLC-2132	0.2563	114
MLC-2171	0.2919	108
MLC-2172	0.2939	111
QYTN-802	0.199	130
QYPDLC-142	0.251	105

The data has been obtained from the Merck and Qingdao datasheets.

### Refractive index measurement

For the measurement of the refractive indexes, we followed the method described in^22^ using an NAR-4T temperature-controlled Abbe refractometer from Atago. In this technique, the molecules of the mixture are aligned perpendicular to the measuring and illuminating prisms of the Abbe refractometer by coating their surfaces with a solution of 1.5 wt% hexadecyltrimethylammonium bromide (HMAB) in methanol. We obtained the two refractive indexes using a polarizing eyepiece. In our reflectometer, we used an incandescent white source and an FB610-10 bandpass filter model from Thorlabs for the measurement of the refractive indexes at ${\lambda }_{R}=613\,{\rm{nm}}$.

### Cell manufacturing

The LC cells were made from 1 × 3 inch UV grade fused silica microscope slides (185~2500 nm) from Advalue Technology. After cutting the slides to 1 × 1 in and thoroughly cleaning them, we created an alignment layer by dip coating. We used two coating materials, PMMA (poly(methyl methacrylate) ${M}_{w}=120000$) from Sigma Aldrich and a polymercaptan compound we obtained from the hardener of the two component epoxy cement from Gorilla. For the dip coating with PMMA, we used a 2 wt% solution in chloroform and a constant speed of $1.7\,{{\rm{mms}}}^{-1}$. For the polymercaptan, we used a 2 wt% solution in butanone and a constant speed of $2\,{{\rm{mms}}}^{-1}$. Once the cell plates were prepared, we used precision microspheres as separators between the plates to create cavities with uniform thickness. For cell gaps below 9 μm, we used monodisperse silica standard microspheres from White House Scientific, and for larger gaps, we used solid soda lime glass microspheres from Cospheric. The bondline for closing the cell was created using a mixture of NOA UVS91 30 wt% and NOA 61 70 wt% from Norland, which was the best solution based on the ease of creating a thin bonding line (the more viscous NOA 61 is better for this) and the capability for withstanding thermal cycles of 110 °C for 2 h (the less viscous NOA UVS91 is better for this). Additionally, both UV adhesives do not interact with the liquid crystal mixtures. The bondline was cured with a 365 LED for 1 min, and the cell was postcured @365 nm in a BIO-LINK Crosslinker BLX-365 UV curing oven for 10 min. Finally, the cell was filled under vacuum through an opening in the bond line, which was sealed after filling.

### Irradiance measurement

To measure the spatial irradiance distribution at the sample plane (see Fig. 2a), we measured the radiant flux during scanning of the beam in steps of 0.5 mm with an optical power meter (PM100 with an S120UV head from Thorlabs) masked with a pinhole of 0.5 mm diameter. From the measured flux and the pinhole area, we estimate the irradiance at every sampling point.

## Supplementary information

Supplementary material.

Supplementary video.

## Data Availability

The datasets generated and analyzed during the current study are available from the corresponding author on reasonable request.
