# Supplementary material for: Reversible Photoalignment of Liquid Crystals: a Path toward the Creation of Rewritable Lenses
Source: Sci Rep. 2020 Apr 1;10:5739. doi: 10.1038/s41598-020-62778-2 (PMC7113309; doi:10.1038/s41598-020-62778-2)
Supplement: Supplementary file 1 — Supplementary material. [file 41598_2020_62778_MOESM1_ESM.docx]

Supplementary Material

Reversible Photoalignment of Liquid Crystals: a Path toward the Creation of Rewritable Lenses

Juan Antonio Quiroga*, Ignacio Canga, José Alonso, Daniel Crespo

Indizen Optical Technologies SL

# Supplementary material

## Supplementary NOTE 1 EPPA in thick cells

In this note, we discuss the recording-erasing process driven by the EPPA effect in thick cells using the same experimental setup described in the main text. Supplementary figure 1a shows two recording cycles for a $d=41.7 \mu m$ cell filled with MLC-2132. For the first recording cycle (in black, labeled $\delta_{1}$ in Supplementary figure 1a), the retardation does not reach zero because the director orientation does not reach the homeotropic state for the whole cell thickness. In this case, the retardation varies between $\delta_{M}=\frac{2\pi}{\lambda_{R}}d\Delta n=101 rad$ and $\delta_{min} =30 rad$. For this sample, the irradiance at the point of interest was $E=119.6 mWcm^{-2}$. For the first recording cycle, we reached the retardation saturation after irradiating the sample for $t_{1}=9870 s$ using 329 exposure steps of $\Delta t=30 s$, with an accumulated exposure of $H_{1}=1180.4 Jcm^{-2}$. After this, we erased the sample by heating it at $T=85 C$ for 20$h$ and started a second recording process. The resulting retardation as a function of exposure is labeled as $\delta_{2}$ in Supplementary figure 1a. For this second cycle, to reach the retardation saturation, we needed a total time of $t_{2}=6990 s$, with a total exposure of $H_{2}=836 Jcm^{-2}$. The first recording $\delta_{1}\left( H \right)$ presents the usual elbow near $550 Jcm^{-2}$, and the second recording $\delta_{2}\left( H \right)$ has no elbow, as occurred in thin cells. For this thick cell, we can observe a final step-like shape due to the action of the magnetic field in the cell volume during the recording. If the magnetic field is removed, a retardation change occurs due to the realignment of the liquid crystal molecules within the cell volume. Moreover, even without the magnetic field, for cells thicker than $40-50 \mu m$, the retardation saturates before it becomes zero. This phenomenon could be produced if both cell surfaces do not reach the same polar angle. In this case, the integrated retardation - see equation (4) in the main text - cannot become zero even if one of the surfaces is in the homeotropic condition.

## Supplementary NOTE 2 EPPA dependence on the of the recording UV beam directionality and the type of alignment layer

In our former experiments, we used a highly directional converging UV beam to obtain large exposures at the cell plane and smaller irradiation times to reach a given exposure. Additionally, the use of alignment layers makes the creation of clean retardation patterns, such as the GRIN lens shown in Supplementary figure 2, possible. However, the same results can be obtained using diffuse light and cells without an alignment layer. To verify this possibility, we performed a recording experiment using a BIO-LINK Crosslinker BLX-365 UV curing oven working at $\lambda=365 nm.$ This type of curing oven generates a uniform nondirectional irradiance distribution at the curing plane. The average measured irradiance at the curing plane was $E=12.4 mW/cm^{2}$. We manufactured two cells using $7 \mu m$ spacers, following the usual method for cell preparation. One of the cells was prepared with a PMMA alignment layer and the other without it, which we denote number 1 and 2, respectively. We filled the cells with MLC-2132, creating a homogeneous planar cell in both cases. The measured thicknesses at the empty cell centers were $d_{1}=8.4 \mu m$ and $d_{2}=7.3 \mu m$. With the cells placed at the oven curing plane, we irradiated the samples for a total time of $t=9.5 h$. During the first 6 h, we irradiated at 1 h intervals, and from this point onward, we used 0.5 h irradiation intervals. After each exposure, we measured the retardation at the same point of the cell off-line. We used different intervals because after 6 h, we reached the elbow for these samples, and from this point onward, the quick retardation change required shorter exposure intervals. For the retardation measurements, we used a model BX53M polarization microscope from Olympus equipped with a Berek compensator calibrated for the $D$ line ($\lambda_{D}=587.6 nm$). At the end of the recording process, we reached the homeotropic state for both cells, with an accumulated exposure of $H=424.1 Jcm^{-2}$. Supplementary figure 1b shows the recorded retardation as a function of the exposure. The measured retardations with and without the alignment layer are labeled $\delta_{1}$and $\delta_{2}$, respectively. In both cases, we obtain a two-regime curve with an elbow similar to the curve shown in figure 3g of the main text. On the one hand, this result indicates that the EPPA effect does not depend on the recording beam directionality but on the accumulated exposure. On the other hand, this result shows that the EPPA depends on the interaction between the liquid crystal and the surface and is not related to the alignment layer interaction with the UV radiation.

## Supplementary NOTE 3 EPPA temporal stability

With respect to the temporal stability, we recorded a GRIN lens in a $51 \mu m$ thick homogeneous planar cell filled with MLC-2132. In Supplementary figure 2a, we show the CDF image of the initial pattern. We monitored the cells at room temperature for 7 days. Supplementary figure 2b-e shows the CDF images of the cell after 1, 2, 5 and 7 days. The CDF images - equation (3) in the main text - are interferograms, for which each fringe is an isoline of the retardation (and therefore reflects the changes in the polar angle). In Supplementary figure 2a, the fringe center corresponds to the zone with smaller polar angles (close to the homeotropic condition), and the fringe pattern lateral areas have larger polar angles (where the configuration is homogeneous). Thus, the images in Supplementary figure 2 depict a negative lens whose effective refraction index is smaller at the center than at the sides. We measured this lens with a deflectometer, obtaining a power of -1 D at the lens center. To check for the temporal evolution of the GRIN lens shown in Supplementary figure 2a, in Supplementary figure 2f, we plot the measured retardations along the white horizontal line drawn in Supplementary figure 2a for all the temporal samples. The EPPA is quite stable for the first 3 days. After this period, a fading process in the retardation begins that can be interpreted as a relaxation of the polar orientation angle from the homeotropic to homogeneous condition. In our experiments, usually after two weeks, the polar angle is almost completely relaxed.

## **Supplementary NOTE 4** EPPA wavelength dependence

In this note, we discuss a qualitative assessment of the wavelength dependence of the EPPA. Experimentally, we observe that above 380 nm, no EPPA effect occurs in the liquid crystals we used, so we centered our study at 326 nm and 365 nm because below 320 nm, our light sources do not have significant radiant flux. We built several MLC-2132 quartz cells using $7 \mu m$ spacers with polymercaptan as an alignment layer. This polymer is known for having small anchoring energies [1], facilitating the initial azimuthal alignment and further observation of the EPPA effect. In quartz cells using polymercaptan as an alignment layer, the MLC-2132 mixture forms homogeneous planar cells. Before irradiation, we azimuthally aligned the director using a magnetic field along the $X$ axis [2]. In this experiment, we used a white light plasma source (HPLS-30-03 from Thorlabs) filtered at 326 nm and 365 nm by two 10 nm half-width bandpass filters from OptoSigma, with references VPF-25C-10-25-32600 and VPF-25C-10-25-36500, respectively. We irradiated the cells until we observed the appearance of the homeotropic state, indicating a total reorientation of the polar angle. The results are summarized in Supplementary table 1, where we show the irradiance, the total time to reach the homeotropic state and the accumulated exposure for the two wavelengths. For the exposure, a factor of $\approx7\times$in the efficiency for $\lambda=326 nm$ versus $\lambda=365 nm$ can be observed. However, the irradiance for $326 nm$is approximately $80\times$ lower than that for $365 nm$. Therefore, we need approximately $10\times$ more irradiation time with $326 nm$ than with 365 nm. This difference is the main reason why we used $365 nm$ for the recording phase of our EPPA experiments. In our case, $365 nm$ is the best compromise in terms of time and exposure efficiency for the light sources we have in our laboratory.

# Supplementary references

1. Oswald, P., Dequidt, A. & Żywociński, A. Sliding planar anchoring and viscous surface torque in a cholesteric liquid crystal. *Phys. Rev. E* **77**, 061703 (2008).

2. Nemoto, F., Nishiyama, I., Takanishi, Y. & Yamamoto, J. Anchoring and alignment in a liquid crystal cell: self-alignment of homogeneous nematic. *Soft Matter* **8**, 11526 (2012).

# Supplementary Figures

| a)    b)   |
| --- |
| Supplementary figure 1. a) Retardation curves for two recording cycles for a $41.7 \mu m$ cell. The recording dynamics for the EPPA is similar to that for the thin cells in the main text. However, the retardation does not become zero, indicating that some volume effect hinders the total reorientation of the director along the cell thickness. b) Retardation evolution with exposure for PMMA-coated ($8.4 \mu m$ thickness, labeled $\delta_{1}$) and uncoated ($7.3 \mu m$ thickness, labeled $\delta_{2}$) quartz cells when using a diffuse UV light source. In both cases, the retardation curve is similar to the first recording of the $5.8 \mu m$ cell shown in figure 3g of the main text, where we used a collimated UV beam. |

| a)  | b)  | c)  | d)  | e) |
| --- | --- | --- | --- | --- |
| f) | | | | |
| Supplementary figure 2. Temporal stability of a lens recorded using the EPPA effect with a measured power of $-1D$. In this case, we used a $51 \mu m$ cell with MLC-2132. a) CDF image of the initial recorded state. b)-c)-d) and e) Images of the CDF pattern after 1, 2, 5 and 7 days, respectively. f) Plot of the retardation variation $\delta\left( x \right)$ along the horizontal white line shown in a) for each temporal sample. The first three days, the recording is quite stable. After this period, the retardation starts fading, indicating a relaxation of the polar angle toward a homogenous condition. | | | | |

| \| $\boldsymbol{\lambda}\left( \boldsymbol{nm} \right)$ \| $\boldsymbol{E}\left( \boldsymbol{mWc}\boldsymbol{m}^{\boldsymbol{-2}} \right)$ \| $\boldsymbol{t (s)}$ \| $\boldsymbol{H}\left( \boldsymbol{Jc}\boldsymbol{m}^{\boldsymbol{-2}} \right)$ \| \| --- \| --- \| --- \| --- \| \| 326 \| 0.329 \| 81000 \| 21.32 \| \| 365 \| 25 \| 6000 \| 150 \| |
| --- | --- | --- | --- | --- | --- | --- | --- | --- | --- | --- | --- | --- |
| Supplementary Table 1 Irradiance, total time and total exposure to reach the homeotropic state for $7 \mu m$ cells with polymercaptan as an alignment layer at 326 and 365 nm. In terms of exposure, irradiation at 326 nm is approximately $7\times$ more efficient than that at 365 nm. However, for our source, the radiant flux is approximately $80\times$ smaller. Therefore, for our sources, the irradiation at 365 nm is $10\times$ faster than that at 326 nm. |
